# Carbon isotope effects in cometabolic oxidation of halogenated organics by a methanotroph

**DOI:** 10.1007/s11356-025-37190-w

**Published:** 2025-11-28

**Authors:** Pratibha Rauniyar, Almog Gafni, Alison Cupples, Anat Bernstein

**Affiliations:** 1 The Albert Katz International School for Desert Studies, The Jacob Blaustein Institutes for Desert Research, Ben-Gurion University of the Negev, Sede Boqer, Israel; 2 The Zuckerberg Institute for Water Research, The Jacob Blaustein Institutes for Desert Research, Ben-Gurion University of the Negev, Sede Boqer, Israel; 3 Department of Civil and Environmental Engineering, Michigan State University, East Lansing, MI USA

**Keywords:** Halogenated groundwater pollutants, Cometabolism, *Methylosinus trichosporium* OB3b, Isotope fractionation, CSIA, AKIE

## Abstract

Cometabolic oxidation is a process in which compounds, including groundwater pollutants such as halogenated aliphatics, are fortuitously oxidized by enzymes with broad substrate specificity. Assessing the magnitude of cometabolic oxidation in contaminated environments is challenging; however, it may be facilitated using Compound-Specific Isotope Analysis. Our former work on the cometabolic oxidation of trichloroethene (TCE) revealed a unique isotope pattern when the process was catalyzed by several methanotrophs rather than by toluene and ammonia oxidizers. In the current work, we aimed to study isotope effects in the cometabolic oxidation of other halogenated compounds of environmental interest by the methanotroph *Methylosinus trichosporium* OB3b. Results showed relatively small carbon isotope fractionation for cis-dichloroethene (cDCE; AKIE = 1.0017 ± 0.0016) and bromoform (BF; AKIE = 1.0020 ± 0.0014), which are similar to formerly determined value for TCE. Larger carbon isotope effects were recorded for chloroform (CF; AKIE = 1.0038 ± 0.0007) and dichloromethane (DCM; AKIE = 1.0043 ± 0.0009), possibly implying a decrease in the role of enzyme binding as a bottleneck for these two. Nevertheless, while DCM presented both relatively large carbon isotope effects and rapid degradation, CF presented relatively large carbon isotope effects but degraded slower than cDCE and TCE. This is possibly related to the reported toxicity of CF’s byproducts, which reduces the overall degradation rate.

**Supplementary Information:**

The online version contains supplementary material available at 10.1007/s11356-025-37190-w.

## Introduction

Halo-organic compounds of anthropogenic origin, such as trichloroethene (TCE) or cis-dichloroethene (cDCE), threaten groundwater quality worldwide (Burri et al. 2019). In contaminated groundwater, these compounds may undergo reductive degradation processes that decrease their concentrations; however, these processes can also lead to the accumulation of transformation products that are of similar or even greater toxicity (e.g. vinyl chloride in the reduction of TCE). Reductive dechlorination involves the sequential formation of less chlorinated transformation products (Holliger et al. 1998) which are easily detected in the environment. In contrast, oxidative processes involving TCE or other halogenated aliphatics often do not lead to the accumulation of easily detected metabolites. Therefore, assessing the magnitude of oxidative processes in the field is challenging. Oxidative degradation of TCE and other halogenated aliphatics may proceed cometabolically. Cometabolic oxidation is a process in which compounds are fortuitously oxidized by enzymes with a broad substrate range, often monooxygenase or dioxygenase (Alexander 1994; Horvath 1972; Wackett 1996). The oxygenase is induced by obligatory growth substrates, such as methane, ethene, ammonium, and aromatic hydrocarbons. With their lower specificity, oxidation of other compounds in addition to the growth substrates may occur (Alexander 1994; Wackett 1996). This can positively reduce the concentrations of groundwater pollutants such as halogenated aliphatic compounds (e.g., (Jesus et al. 2016)) or 1,4-dioxane (e.g. (Chen and Hyman 2023)).

Much research has been done on the cometabolic oxidation of the environmental contaminant trichloroethene (TCE). The first TCE cometabolizing bacteria to be discovered were the methanotrophs (Wilson and Wilson 1985). Methanotrophs utilize two types of methane monooxygenases (MMOs): soluble MMO (sMMO), which is only expressed by a subset of methanotrophs, and the membrane-bound particulate MMO (pMMO), which is more ubiquitous. These two types of MMOs are genetically unrelated, and their expression can be regulated by copper concentrations (Semrau et al. 2010). Other TCE cometabolizing bacteria were later discovered, including bacteria grown, for example, on toluene, ammonia, propane, etc. (Arp et al. 2001).

Assessing cometabolic oxidation of TCE in the environment is challenging due to the lack of easily detectable indicative products. Compound-Specific Isotope Analysis (CSIA) may bridge this gap and provide a unique insight into the processes that the compound followed since being released to the environment (Elsner et al. 2012; Hunkeler et al. 2008; Kuntze et al. 2020). To use this tool, isotope effects must be determined for the studied processes. In the case of TCE’s cometabolic oxidation, a C = C bond is initially cleaved, forming an epoxy intermediate (Fox et al. 1990, Shuying and Wackett 1992). It may, therefore, be expected that the process would be accompanied by a small (secondary) chlorine isotope effect, along with a strong (primary) carbon isotope effect, as reported for its abiotic oxidation by permanganate (Doğan-Subaşı et al. 2017). This enrichment trend differs from the isotope enrichment trend documented for reductive dehalogenation (Cretnik et al. 2013) and may potentially enable differentiation between the two processes in contaminated groundwater. Indeed, our previous work on TCE cometabolic oxidation by toluene and ammonia oxidizers confirmed the expectation of a secondary chlorine isotope effect along with a primary carbon isotope effect. However, an unexpectedly different δ^13^C vs. δ^37^Cl isotope trend was identified when the process was catalyzed by methanotrophs (Gafni et al. 2020a, b; Gafni et al. 2018). The different pattern of methanotrophs was explained by slow rate limiting steps prior to catalysis. Mass transfer limitations (or bottlenecks) increase the enzyme’s “commitment to catalysis” (Northrop 1981), which, in turn, decreases the enzyme’s selectivity towards lighter isotopes. Different rate limiting steps prior to catalysis can theoretically be considered as potential bottlenecks (e.g., slow transfer through the cell membrane (Nijenhuis et al. 2005; Renpenning et al. 2015) or limited substrate’s concentration (Ehrl et al. 2018)). In the case of cometabolic oxidation by methanotrophs, substrate binding was suggested, based on former studies of ^1^H/^2^H kinetic isotope effects with purified or mutated soluble methane monooxygenases (sMMO) (Ambundo et al. 2002; Brazeau et al. 2001; Nesheim and Lipscomb 1996; Valentine et al. 1999). The reason why substrate binding is rate limiting in methanotrophs was first linked to the substrate’s size sensitivity (Brazeau et al. 2001) and later extended to a more complex interplay between to the substrate’s size and polarity (Ambundo et al. 2002; Kopp and Lippard 2002).

While systematic studies were carried out for purified enzymes and reported above, more arbitrary work was done with living methanotroph cells, and especially with compounds that are relevant as environmental pollutants. For example, in one study, the cometabolic oxidation of *trans*-DCE by strain OB3b resulted in a relatively large carbon isotope enrichment factor (ε in Eq. 1) of −6.7‰ (Brungard et al. 2003). In a different study, the cometabolic oxidation of the less chlorinated molecule vinyl chloride by the same strain was accompanied by a carbon isotope enrichment factor of −3.2‰ (Chu et al. 2004). Since the experimental systems differed between the studies (e.g., strains used, cell densities, incubation conditions, etc.), it becomes difficult to draw a general rule for an expected carbon isotope effect for these compounds catalyzed by methanotrophs. A more systematic study, in this respect, is needed. This work, therefore, aimed to study the isotope effects and degradation kinetics for selected halogenated methanes and ethylenes. Specifically, comparisons between C1 vs. C2 compounds, tri- vs. di-halogenated, and brominated vs. chlorinated compounds are presented.

## Materials and methods

### Experimental setup

The pure strain of the methanotroph *Methylosinus trichosporium* OB3b was cultivated and used for all experiments. This strain was kept as frozen stock cultures at −80 ⁰C. The culture was cultivated in a liquid medium (DSMZ Medium 921 (DSMZ 2007)) without amendment of CuSO_4_ to induce the expression of sMMO (Nielsen et al. 1997). All experiments were accompanied by negative controls (no bacteria).

For all experiments, dense cultures were prepared, as detailed below. Experiments were conducted with resting cells after harvesting a large volume of dense cultures, which was resuspended in a fresh growth medium that contained the studied compounds without the addition of methane. Incubation lasted up to 24 h.

Dense cultures were prepared as follows: Cultures were first grown in replicate 15 mL of liquid growth media (DSMZ Medium 921; (DSMZ 2007)) in 60 mL serum bottles. After approximately 48 h, the duplicates (30 mL in total) were transferred to 1 L glass bottles containing 300 mL of liquid growth media and capped with a Teflon-lined septa. In both cases, as a carbon source, methane was amended to the gas phase in a volume of 20–50% of the total gas phase (Maxima, 99.95%). Cultures were incubated in the dark at 30 °C on an orbital shaker (~ 120 rpm). Before starting the experiments, cultures were aerated, resealed, and amended daily with a fresh carbon source using a syringe until a dense culture in the logarithmic phase (optical density at 600 nm up to 0.8) was achieved. The cultures were harvested by centrifugation at ~ 10k rpm for 15 min and resuspended in fresh growth medium and used for the experiments.

Kinetic experiments were performed to determine the cometabolic oxidation rates of cDCE, DCM, chloroform, and bromoform simultaneously in separate flasks, ensuring identical cell densities and growth conditions. Experiments were conducted in autoclaved 20-mL headspace (HS) vials (screw thread vial capped with 18-mm screw cap with a Teflon-lined septa, Thermo Fisher Scientific). First, the dense harvested culture was equally divided between all vials in a volume of 0.25–1.0 mL (depending on the volume of the dense harvested culture, which varied between experiments). The abiotic controls were amended with 0.25–1.0 mL of sterile growth media instead of dense harvested culture. Time zero vials were also amended with ~ 3 drops of 85% phosphoric acid. Then, the growth medium was added to all vials to a final volume of 10 mL. The growth medium contained the dissolved halogenated substrate at an initial concentration range of 3.3–14 mg/L in different experiments (Table S2), resazurin (1 mg/L) as a redox indicator to verify that aerobic conditions are maintained, and potassium formate (1682 mg/L) as a reducing source for methanotrophic cultures. The experimental medium for each halogenated compound stirred in capped bottles (1 L) overnight on a magnetic stirrer prior to inoculation to verify full dissolution of the compounds. After adding the growth medium, HS vials were immediately capped with metal screw caps with a Teflon-lined septa. Vials were incubated upside down in the dark on an orbital shaker at 120 rpm at 30 ⁰C. Sampling was done sacrificially, by stopping the degradation process in two or three replicate vials (depending on the experiment) at selected time points. Degradation was stopped by adding ~ 3 drops of 85% phosphoric acid (achieving pH ≤ 2). Acid was added with a syringe by piercing the septa with a thin needle (0.60 × 32 mm). The experiment was accompanied by abiotic controls (culture not added) at time zero and the final time to account for evaporation or abiotic degradation processes. The samples were analyzed by GC-FID to quantify the concentrations of the respective halogenated substrates in the vials.

The experiments for determining isotope effects followed the same procedure as the kinetic experiments, although for each compound at different time periods rather than simultaneously. Preliminary experiments were conducted in serum bottles rather than in HS vials. In this case, 1.5 mL of the concentrated bacterial culture was transferred into 60-mL autoclaved serum bottles for initiating the experiments. Growth medium (15 mL) was added to each of the bottles and immediately crimped with a Viton septa (20 mm Straight Plug OV P/N 13235). In later experiments, similarly to the kinetic experiments, 0.25–1.0 mL of the concentrated culture was transferred into 20-mL autoclaved HS vials, along with fresh growth medium, to a final liquid volume of 10 mL. Sampling was done sacrificially, by stopping the degradation process in two replicate vials at selected time points.

### Analytical methods

#### Chemical analysis

Halogenated compounds were quantified using Gas Chromatograph coupled to a Flame Ionization Detector (GC-FID) (Thermo Scientific TRACE 1300 Series and a TRACE 1310 Auxiliary Oven). For peak separation, a DB5 column was used (30 m × 0.25 mm × 0.25 µm; RESTEK). Headspace injection was carried out by a TriPlus RSH (Thermo Scientific) auto sampler. The 20-mL experimental HS vials were agitated at 50 °C for 5 min before injection. The injector was maintained at 250 °C, flow velocity was 1 mL/min with a split ratio of 10 mL/min. The oven started at 40 °C (1 min), followed by a ramp of 20 °C/min to 60 °C and a second ramp of 45 °C/min to 230 °C, which was held for 1 min.

#### Isotope analysis

Carbon isotope analysis was carried out by a GC-IRMS (Trace Ultra GC hyphenated to a Delta V Plus; Thermo Scientific). The instrument was equipped with a commercial Thermo combustion unit that was operated at 1000 ⁰C. Peak separation was achieved using a Rxi®−624Sil MS GC column (30 m × 0.25 mm × 1.4 µm; RESTEK). Headspace injection was carried out by a TriPlus RSH (Thermo Scientific) auto sampler. Then, 20-mL HS vials that contained 10 mL of the liquid sample were agitated at 50 ⁰C (DCM) or typically 70 ⁰C (CF, BF, cDCE) for 5 min. The injector was held at 250 °C with column flow of 1.4 mL/min and a split flow of 14 mL/min. The oven temperature program differed for the different analytes. For DCM, oven temperature started at 35 °C held for 1 min, followed by a temperature ramp of 30 °C/min to 120 °C, a second ramp of 50 °C/min to 270 °C, which was held for 2 min. For the other analytes, oven temperature started at 40 °C, followed by a temperature ramp of 50 °C/min to 180 °C, a second ramp of 50 °C/min to 270 °C, which was held for 3 min.

Three CO_2_ reference peaks, calibrated to the international standards (VPDB), were introduced to the IRMS at the beginning and at the end of each run to determine the sample’s δ^13^C.

#### Cell quantification

In the kinetics experiment, *M. trichosporium* OB3b cells were quantified using flow cytometry. SYBR Green, a DNA-binding dye, has been widely used to stain viable cells in both flow cytometry and fluorescence microscopy since the early 2000 s (Barbesti et al. 2000; Davey 2011) and was employed in this study for cell staining. Samples of the harvested bacterial culture were collected (1.7 mL) and fixed with 0.2% glutaraldehyde (Sigma Aldrich) for 10 min and stored at − 80 °C. Prior to counting, samples were fast-thawed at 37 °C, and bio-aggregates were dismantled using 5 mM of EDTA and sonication. Samples were stained with 0.5 nM of SYBR® Green II RNA Gel Stain (Thermo Fisher Scientific S7564) in the dark for 15 min. Samples were analyzed with an Attune-Next acoustic focusing flow cytometer (Applied Biosystems) equipped with a syringe-based fluidic system at 408 and 488 nm wavelengths at a flow rate of 25 µL/min. Beads (nominal size 0.93 µm) (Polysciences) were used as a size standard.

### Calculations

The change in the isotope ratio, R/R_0_, along the extent of degradation, *f*, is described by the Rayleigh equation:

1 $$In\left(\frac R{R_0}\right)=\varepsilon\cdot\ln f$$

Using the delta notation, when δ is much smaller than 1, the above equation can be expressed in a simplified form as (Aelion et al. 2009):

2 $$\delta{}^{13}C=\delta{}^{13}C_0+\varepsilon\cdot lnf$$

The isotope enrichment factor, ε_bulk_, was determined as the slope of the isotope composition, δ^13^C, versus the natural logarithm of the degradation extent, f. The 95% confidence interval was calculated for the slope of the regression line and sets the uncertainty for the reported ε values below.

Based on the experimentally derived ε_bulk_, the apparent kinetic isotope effect (AKIE) was calculated as (Elsner et al. 2005):

3 $$\text{AKIE}=\frac{1}{1+\left( \frac{z\times n }{x}{\upvarepsilon }_{\text{bulk}}\right)}$$

where *n* is the number of carbon atoms in the molecule, *x* is the number of carbon atoms at a reactive site, and *z* is the number of carbon atoms at reactive sites that are in intra-molecular competition. In the case of halogenated methanes, *n*, *x*, and *z* are all equal to 1. In the case of cDCE, *n* and *x* are equal to 2, while *z* is equal to 1.

Pseudo-first-order rate model was applied to quantitatively describe the cometabolic oxidation as (Alvarez-Cohen and Speitel Jr 2001, Jesus et al. 2016):

4 $$r_c=-k_1\cdot X\cdot S_c$$

where r_c_ is the rate of the reaction (mg L^−1^ h^−1^) k_1_ is the pseudo-first-order rate constant (L cells^−1^ h^−1^), *X* is the cell concentration (cells L^−1^), and S_c_ is the substrate concentration (mg L^−1^). Since experiments were conducted for all five substrates simultaneously with identical cell concentrations, we report an apparent rate constant for the given experimental system, k’ (h^−1^), as:

5 $$r_c=-k'\cdot S_c$$

## Results and discussion

### Carbon isotope effects in the cometabolic oxidation of the tested compounds

**Fig. 1:**
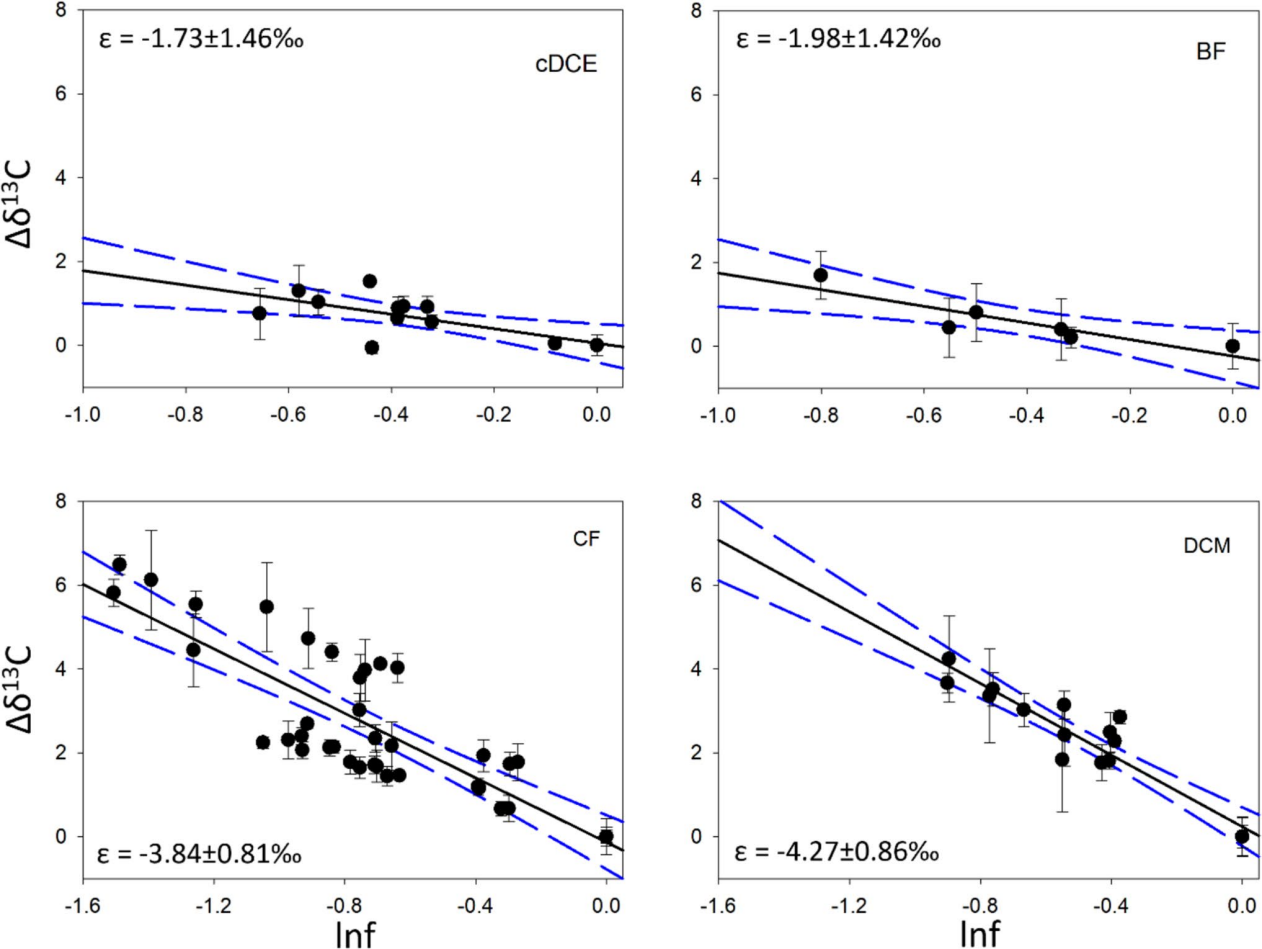
Rayleigh plots for the tested compounds. Error bars represent the standard deviation between duplicate analyses of the samples by IRMS. The plots include data from several independent experiments. Results for each individual experiment are presented in the Supplementary Information (SI)

The current study shows a relatively small carbon isotope effect for the halogenated ethene, cDCE, and for the brominated methane, BF, with ε_bulk_ of −1.86 ± 1.62‰ (AKIE = 1.0017 ± 0.0016) and −1.98 ± 1.42‰ (AKIE = 1.0020 ± 0.0014), respectively. These values are not significantly different from values that were determined for the cometabolic oxidation of TCE by strain OB3b, and which were previously defined as secondary isotope effects (Gafni et al. 2020a, b). Stronger isotope effects were observed for the two chlorinated methanes, CF (ε_bulk_ = −3.84 ± 0.81‰; AKIE = 1.0038 ± 0.0007) and DCM (ε_bulk_ = −4.27 ± 0.86‰; AKIE = 1.0043 ± 0.0009) (Fig. 1). These larger values enable more sensitive assessments of the process in polluted groundwater (Gafni et al. 2020a, b).

In comparing the AKIE of halogenated methanes to cDCE, we must consider that different bonds are involved. While in oxidation of BF, CF, and DCM a C-H bond is being cleaved, forming a hydroxylated product, in cDCE oxidation a C = C bond is initially cleaved to form an epoxide intermediate. Therefore, it is questionable whether the observed differences stem from a different reaction mechanism or from the distinct roles played by the bottlenecks. Nevertheless, previous studies indicate that, despite differences in mechanism, the unmasked isotope effects in both cases are expected to fall within the same range. In the case of C = C bond oxidation, the abiotic permanganate oxidation of TCE presented ε values of −21.4 to −26.8‰, implying an AKIE of 1.021 to 1.027 (Hunkeler et al. 2003; Poulson and Naraoka 2002). The highest ε values of TCE cometabolic oxidation for living cells were reported for an ammonia oxidizing mixed culture and were −24.8 ± 5.2‰ (Gafni et al. 2020a, b), which falls in this range. For C-H bonds, the theoretical limit (Streitwieser Semiclassical Limit) for carbon KIE in C-H bond cleavage is 1.021 (Elsner et al. 2005). In experimental systems, microbial methane oxidation by sMMO was accompanied by an AKIE of 1.027 by a mixed culture (Kinnaman et al. 2007), and of 1.015–1.029 in methane oxidation by different types of methanotrophs (Feisthauer et al. 2011).

### Degradation kinetics

Degradation kinetics by resting cells were studied for five compounds simultaneously, using the same bacterial density and identical growth stage. Therefore, the results for each substrate can be considered comparable to the others. Cells were counted by flow cytometer yielded a cell density of 4.7 × 10^7^ cells/mL.

**Fig. 2:**
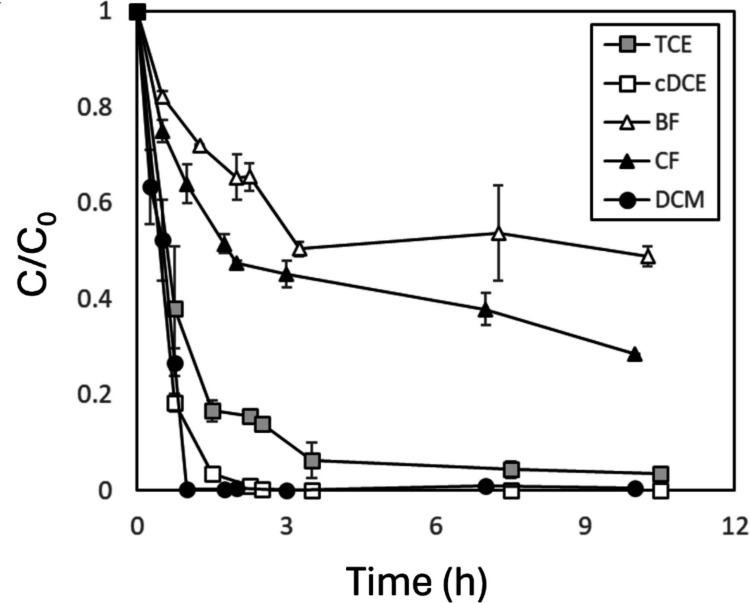
Degradation extent (C/C_0_) of the tested halogenated compounds. Error bars represent the standard deviation between duplicate samples at each time point

The results followed first-order kinetics at early time points, with the reaction slowing to a complete stop within hours (Fig. 2). This is expected, as the experiments were conducted with resting cells; the bacteria were incubated without the growth substrate (methane) when the experiment began. Previous studies have shown that with time, the enzymes gradually lose their activity and the impact of byproduct toxicity gradually increases (Alvarez-Cohen and Speitel Jr 2001; Kim et al. 2020). First-order rate constants were calculated for the first two hours. The results indicate a decrease in cometabolic oxidation rate in the order DCM > cDCE > TCE > CF > BF, with apparent pseudo-first-order rate constants of 3.79, 2.13, 0.96, 0.39, and 0.22 h^−1^, respectively (Fig. S3).

In comparing tri-halogenated and di-halogenated compounds, the less chlorinated compounds showed enhanced degradation. This is in agreement with previous studies that showed the general trend of shorter half-lives for lower substituted halogenated ethenes by different cultures (Jesus et al. 2016) and methanes by OB3b (Bartnicki and Castro 1994; Jesus et al. 2016). In comparing brominated and chlorinated compounds, CF presented a somewhat more enhanced degradation than BF. The more rapid degradation of CF, relative to BF, contradicts a previous study that showed the opposite (Bartnicki and Castro 1994) and may possibly result from small differences between the experimental systems. In comparing C1 and C2 compounds, the halogenated ethenes TCE and cDCE presented more rapid degradation than CF but slower than DCM. Our results yielded a first-order rate constant of CF that is 40% of TCE’s, and is consistent with previously reported range of 30–60% (Alvarez-Cohen and Speitel Jr 2001). Previous studies have linked the reaction slowdown to the formation of toxic byproducts, which was shown to be more significant in CF than in TCE (Alvarez-Cohen and McCarty 1991).

Other experiments are presented in the SI in which the bacteria were not quantified, but the general trends are similar: DCM was always the most rapidly degraded, and halogenated ethenes were always more rapidly degraded than trihalogenated methanes. However, some small discrepancies were observed in comparing BF and CF and in comparing cDCE and TCE.

### Comparing isotope effects and degradation kinetics trends

It may be anticipated that the less substrate binding to the enzyme acts as a bottleneck, the more rapidly the compound degrades and the larger the isotope effects are. Indeed, former studies comparing methane and ethane (Brazeau et al. 2001) or CH_3_NO and CH_3_CN (Ambundo et al. 2002) have shown correlation between the degradation rate and isotope effects. The results of the current study show that larger carbon isotope effects are not necessarily observed for the more rapidly degrading substrates. While larger isotope effects are observable for CF than for TCE and cDCE, more rapid degradation of TCE and cDCE than of CF is consistently shown (Fig. 1 and S1). This observation can be attributed to the toxicity of CF metabolites such as phosgene, which are known to reduce degradation rates, as reported by Alvarez-Cohen and McCarty (1991). In the case of byproduct toxicity, the microbial population gradually loses its ability to catalyze the reaction. This leads to a decrease in rate, which is not related to a decrease in bioavailability and, therefore, does not lead to an increase in commitment to catalysis. Thus, this slowdown in the reaction does not influence the isotope effect.

### Environmental significance

**Fig. 3:**
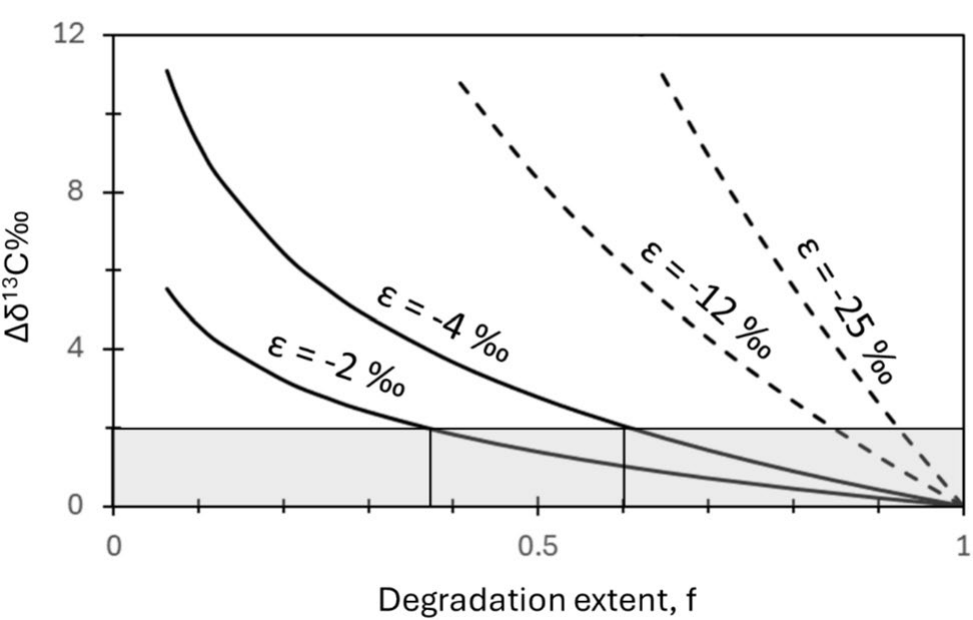
Shift in δ^13^C of cDCE and BF, using a rounded average ε of −2‰, and for CF and DCM, using a rounded average ε of −4‰. Former data for TCE oxidation are represented by dashed lines, with toluene oxidizers using a rounded average ε of −12‰ and ammonia oxidizers using a rounded average ε of −25‰

Identifying degradation in the field requires a significant difference in the spatially measured isotope composition of the target compound, with a recommended threshold of 2‰ (Hunkeler et al. 2008). Advanced degradation of cDCE and BF by methanotrophs would be necessary for shifts of ≥ 2‰ to become detectable. Using a rounded average of ε = −2‰ for cDCE and BF, compared to a rounded average of ε = −4‰ for CF and DCM, suggests that degradation of more than 70% is required for CF and DCM, whereas only 40% degradation is needed for cDCE and BF to confidently identify degradation (Fig. 3). In comparison, previous studies on TCE cometabolic oxidation by toluene and ammonia oxidizers reported significantly larger primary carbon isotope effects (Gafni et al. 2020a, b; Gafni et al. 2018), enabling the process to be tracked at earlier stages of degradation (Fig. 3).

## Conclusions

Small carbon isotope effects are observed during cometabolic oxidation of cDCE and BF by the sMMO-expressing methanotroph *Methylosinus trichosporium* OB3b. In contrast, larger carbon isotope effects are observed for CF and DCM which may suggest a decrease in the role of enzyme binding as a bottleneck for these compounds.Notably, while DCM showed both relatively large carbon isotope effects and rapid degradation, CF exhibited large isotope effects but degraded more slowly than cDCE and TCE. This combination of a larger isotope effect and slower degradation for CF may be attributed to product toxicity.

Isotope analysis is a valuable tool for studying the environmental fate of organic compounds from both natural or anthropogenic origin. This study shows that, similar to TCE, the cometabolic oxidation of cDCE and BF by methanotrophs is associated with low carbon isotope effects, which may limit the applicability of isotope analysis for tracking their in-situ transformation. This tool is of greater potential when CF and DCM are of concern following their larger carbon isotope effects.

## Supplementary Information

Below is the link to the electronic supplementary material.

ESM 1 Supplementary Material 1 (DOCX 323 KB)

## Data Availability

Data will be available on request.
