# Supplementary material for: Carbon isotope effects in cometabolic oxidation of halogenated organics by a methanotroph
Source: Environ Sci Pollut Res Int. 2025 Nov 28;32(51):29028–36. doi: 10.1007/s11356-025-37190-w (PMC12717205; doi:10.1007/s11356-025-37190-w)
Supplement: Supplementary file 1 — Supplementary Material 1 (DOCX 323 KB) [file 11356_2025_37190_MOESM1_ESM.docx]

*Supporting Information*

Carbon Isotope Effects in Cometabolic Oxidation of Halogenated Organics by a Methanotroph

Pratibha Rauniyar^1,2^, Almog Gafni^2^, Alison Cupples^3^, Anat Bernstein^2, *^

^1^ The Albert Katz International School for Desert Studies, The Jacob Blaustein Institutes for Desert Research, Ben-Gurion University of the Negev, Sede Boqer, Israel

^2^ The Zuckerberg Institute for Water Research, The Jacob Blaustein Institutes for Desert Research, Ben-Gurion University of the Negev, Sede Boqer, Israel

^3^ Department of Civil and Environmental Engineering, Michigan State University, East Lansing, MI, USA

Table S1. Molecular weight (ME), average carbon isotope enrichment factor (ε_bulk_), calculated average AKIE, and isotope enrichment factor, ε, for the different experiments

| Compound | MW (gr/mol) | ε_bulk_ (‰) | AKIE | ε_bulk_ (‰), distinct experiments |
| --- | --- | --- | --- | --- |
| TCE* (former study, (Gafni et al., 2020)) | 131.4 | -2.4±0.7 | 1.0024±0.0007 | - |
| cDCE | 97.0 | -1.73±1.46 | 1.0017±0.0016 | Exp. 1: -0.93±1.90  Exp. 2: -2.19±1.32 |
| BF | 252.7 | -1.98±1.42 | 1.0020±0.0014 | Exp. 1: -1.98±1.42 |
| CF | 119.4 | -3.84±0.81 | 1.0038±0.0007 | Exp. 1: -3.93±0.57  Exp. 2: -3.42±-2.65  Exp. 3: -2.36±-0.36  Exp. 4: 3.91±-1.47 |
| DCM | 84.9 | -4.27±0.86 | 1.0043±0.0009 | Exp. 1: -6.05±-1.10  Exp. 2: -4.48±-0.68  Exp. 3: -3.92±-4.11 |

Table S2. Initial concentrations in kinetic experiments (mg/L). Results are presented in Fig. 2 and Fig. S2 (A-C)

| Substrate | Fig. S2, A | Fig. S2, B | Fig. S2, C | Fig. 2 |
| --- | --- | --- | --- | --- |
| DCM | 8.3 | NQ | 7.0 | 3.7 |
| BF | 8.5 | 7.2 | 7.4 | 3.3 |
| CF | 12.9 | 9.6 | 7.7 | 8.2 |
| cDCE | - | NQ | 7.1 | 6.4 |
| TCE | - | NQ | NQ | 9.9 |

NQ: Not Quantified

Fig. S1. Rayleigh plots. Distinct experiments marked by different symbols


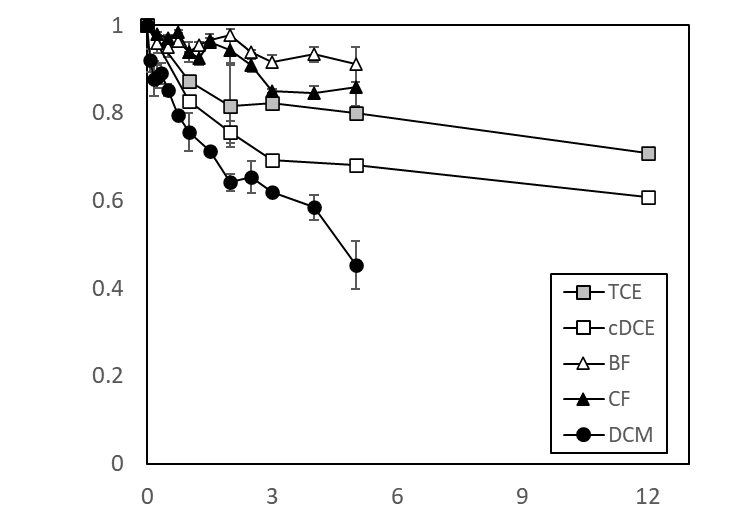


**C**

C/C_0_

Time (h)


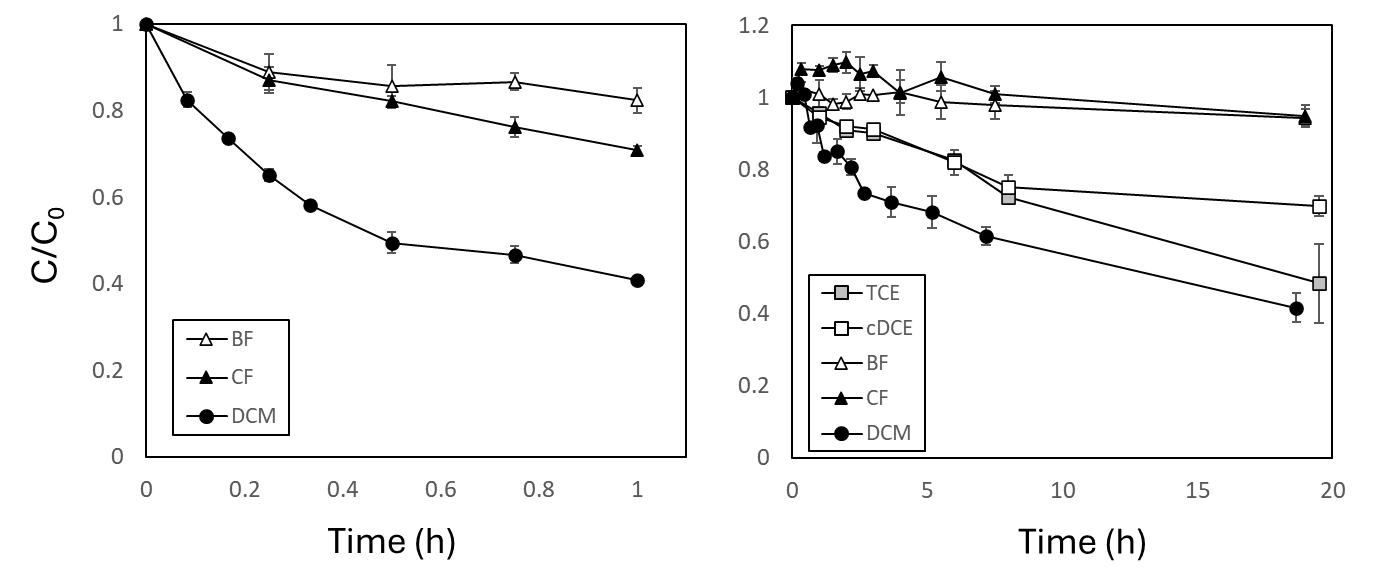


**A**

**B**

Fig. S2. Degradation kinetics in preliminary experiments in which bacteria were not counted (A-B) and in with 9.9 × 10^6^ cells mL^-1^ (C). Duplicate vials were sacrificed at each timepoint, except for (A), where triplicate vials were sacrificed. Error bars represent the standard deviation between replicates.


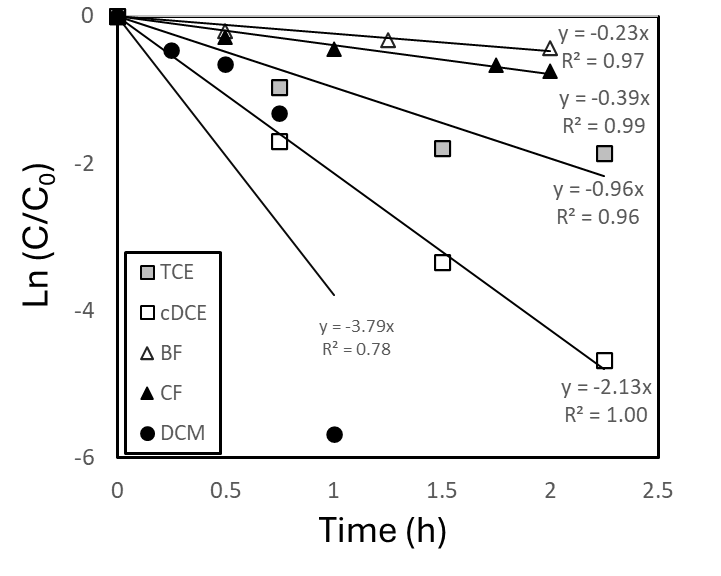


Fig. S3. First order degradation for the first two hours. For DCM, the degradation halted after one hour, and the rate constant was calculated for the first hour (data of Fig. 2).


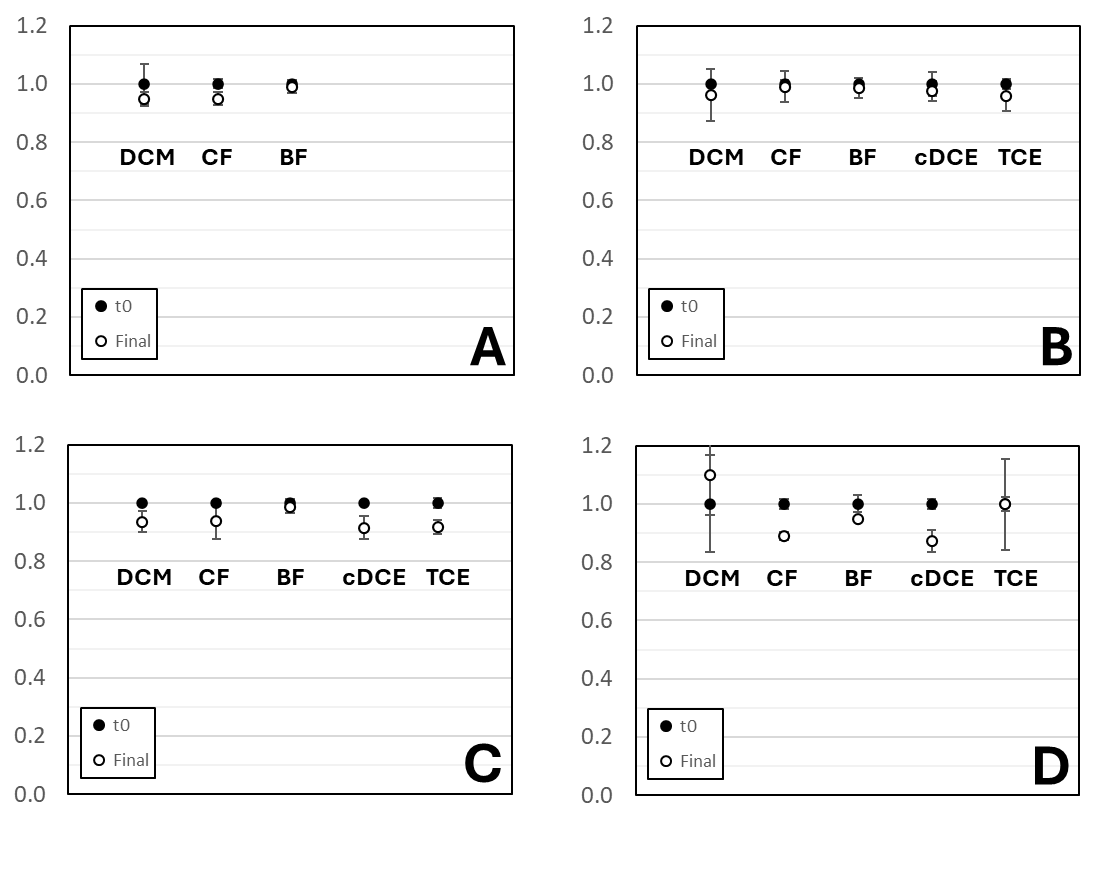


Fig. S4. Triplicate control vials with different analytes were sacrificed (acidified) at the beginning of the experiment ("t0") and in triplicate at the end of the experiment ("Final"). Peak areas of the control vials were measured by GC/FID and are reported here as an average relative to the average of t0 controls. Error bars represent the standard deviation of the triplicate controls for each time point. Panels A-C correspond to panels A-C in Fig. S2, while panel D corresponds to Fig. 2.

Literature

Gafni, A., Gelman, F., Ronen, Z., Bernstein, A., 2020. Variable carbon and chlorine isotope fractionation in TCE co-metabolic oxidation. Chemosphere 242, 125130.
